# Short-term Responses of *Posidonia australis* to Changes in Light Quality

**DOI:** 10.3389/fpls.2017.02224

**Published:** 2018-01-17

**Authors:** Simone Strydom, Kathryn M. McMahon, Gary A. Kendrick, John Statton, Paul S. Lavery

**Affiliations:** ^1^Centre for Marine Ecosystems Research, School of Science, Edith Cowan University, Joondalup, WA, Australia; ^2^Western Australian Marine Science Institution, The University of Western Australia, Crawley, WA, Australia; ^3^School of Biological Sciences, Faculty of Science, The University of Western Australia, Nedlands, WA, Australia; ^4^Centro de Estudios Avanzados de Blanes, Consejo Superior de Investigaciones Científicas, Blanes, Spain

**Keywords:** light quality, light quantity, photomorphogenesis, seagrass, seedlings

## Abstract

Seagrass meadows are highly productive ecosystems that provide ecosystem services to the coastal zone but are declining globally, particularly due to anthropogenic activities that reduce the quantity of light reaching seagrasses, such as dredging, river discharge and eutrophication. Light quality (the spectral composition of the light) is also altered by these anthropogenic stressors as the differential attenuation of wavelengths of light is caused by materials within the water column. This study addressed the effect of altered light quality on different life-history stages of the seagrass *Posidonia australis*, a persistent, habitat-forming species in Australia. Aquarium-based experiments were conducted to determine how adult shoots and seedlings respond to blue (peak λ = 451 nm); green (peak λ = 522 nm); yellow (peak λ = 596 nm) and red (peak λ = 673 nm) wavelengths with a control of full-spectrum light (λ = 400 – 700 nm, at 200 μmol photons m^-2^ s^-1^). *Posidonia australis* adults did not respond to changes in light quality relative to full-spectrum light, demonstrating a capacity to obtain enough photons from a range of wavelengths across the visible spectrum to maintain short-term growth at high irradiances. *Posidonia australis* seedlings (<4 months old) grown in blue light showed a significant increase in xanthophyll concentrations when compared to plants grown in full-spectrum, demonstrating a pigment acclimation response to blue light. These results differed significantly from negative responses to changes in light quality recently described for *Halophila ovalis*, a colonizing seagrass species. Persistent seagrasses such as *P. australis*, appear to be better at tolerating short-term changes in light quality compared to colonizing species when sufficient PPFD is present.

## Introduction

Terrestrial plants detect the light environment and modulate growth and development according to both light quality (the composition of the wavelength-specific radiation within the visible spectrum 400 – 700 nm) and light quantity (Photosynthetic Photon Flux Density, or PPFD) (Fankhauser and Chory, 1997; Whitelam and Halliday, 2008). Light is sensed by photoreceptor proteins that signal transduction cascades. These can lead to a range of physiological, growth and morphological responses influenced by specific wavelengths of light received. The setting of circadian rhythms, flower induction, seed germination, photosynthesis, adult and seedling growth are all influenced by specific wavelengths of light (Fankhauser and Chory, 1997; Chen et al., 2004; Whitelam and Halliday, 2008). In the marine environment, light quality has also been found to be important for marine angiosperm (seagrass) life history processes (Strydom et al., 2017), but information on the influence of specific wavelengths of light for most species is limited (York et al., 2016).

**FIGURE 1:**
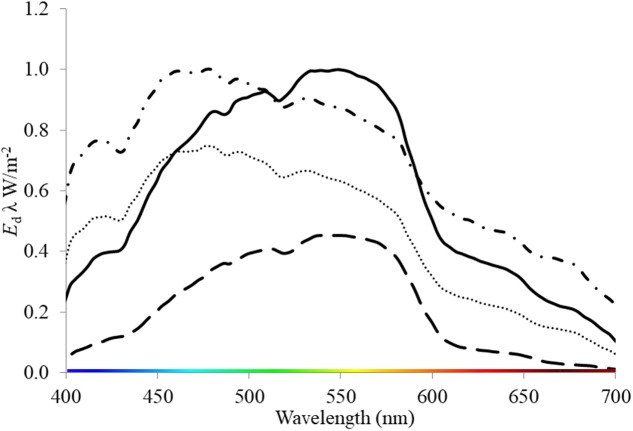
Downwelling irradiance (*E*_d_ λ W/m^-2^) measured underwater at locations where seagrass meadows are present: oceanic, 2 m depth (dash-dot line) and 3.7 m depth (dotted line); and within a dredge plume site at 3 m depth (solid line) and at 7 m depth (dash line). These data were collected by S. Strydom and M. Slivkoff using an underwater hyperspectral radiometer (In-situ Marine Optics Pty Ltd.) in West Australian Coastal Waters (Garden Island and Woodman Point).

Seagrasses are a polyphyletic group of marine angiosperms that evolved from monocotyledonous flowering plants ∼ 85 MY ago (Les et al., 1997). They provide significant ecosystem functions and services in shallow coastal environments globally (Orth et al., 2006). Degradation in water quality (caused by a range of anthropogenic activities, i.e., eutrophication, sediment loading and dredging) that alters light throughout the water column is highlighted as a major contributor to global seagrass loss (Erftemeijer and Lewis, 2006; Waycott et al., 2009; Marbà et al., 2014) and therefore the loss of these functions and services. These activities have the dual effect of reducing the PPFD and altering the spectral quality of light. For example, suspended sediments produced by dredging increase the attenuation of light within the water column (Kirk, 1994; Longstaff and Dennison, 1999; Erftemeijer and Lewis, 2006) and shift the quality of light toward green or yellow wavelengths (**Figure 1**). Light quality also changes naturally with depth as red light is attenuated by water, thus blue light dominates at depth i.e. >10 m (Kirk, 1994). Additionally, wavelengths present within the water column can vary among locations of the same depth depending on particulates and dissolved components of water (**Figure 1**, Strydom et al., 2017). For example, chromophoric dissolved organic matter (CDOM) expelled from rivers and estuaries strongly absorbs short-wave radiation, thus leading to a yellow and red-shifted light field in shallow coastal and estuarine waters (Kirk, 1994).

Seagrass species have similar response mechanisms to reduced PPFD (Ralph et al., 2007; McMahon et al., 2013). For the majority of seagrass species a reduction in PPFD can result in physiological adjustments of photosystems for efficient capture and utilization of available light, remobilisation of carbohydrates as energy reserves to support biomass and reduction in growth to maintain a positive carbon balance during periods of low photosynthetic activity (McMahon et al., 2013). While the timing of responses are species-specific, generally they can be related to their growth strategy. For example, fast-growing “colonizing” species (e.g., *Halophila ovalis*) are negatively and rapidly impacted by light reductions (Longstaff and Dennison, 1999) while “persistent” slow-growing species (e.g., *Posidonia* spp.) have similarly rapid photo-physiological responses but display slower growth and morphological responses due to the buffering role of substantial carbohydrate reserves within their rhizomes (Collier et al., 2009). Therefore, it is likely that as a persistent species, *P. australis* may respond more slowly to altered light quality compared to colonizing species. Some examples of the responses of *Posidonia* spp. to reductions in light availability include significantly lower leaf growth rates and shoot density in *P. australis* (Fitzpatrick and Kirkman, 1995) and reduced leaf density and leaf production in *P. sinuosa* (Gordon et al., 1994). More recently, *P. oceanica* photosynthetic rates (measured as the relative electron transport rate rETR_max_) were significantly lower under reduced light compared to ambient light levels (Hendriks et al., 2017).

The effect of light quality (specific wavelengths of light) on growth of adult plants has recently been reported for *H. ovalis*, where monochromatic blue, yellow and green light negatively impacted below-ground productivity (Strydom et al., 2017). For seagrass seeds and seedlings, contrasting responses to different wavelengths of light have been reported for different species. In *Thalassia hemprichii*, blue light stimulated seedling growth (at 50 μmol photons m^-2^ s^-1^) (Soong et al., 2013), whereas in *H. ovalis*, red light enhanced seed germination and seedling survival (at 200 μmol photons m^-2^ s^-1^) (Strydom et al., 2017). These differing responses to light quality may reflect differences in photoreceptor composition among species. Photoreceptors are well defined in terrestrial angiosperms: phytochromes efficiently absorb red and far red light; cryptochromes, phototropins and the LOV/F-box/Kelch- domain proteins mainly absorb blue and green light; while the photoprotection photoreceptor UVR8 senses UV-B light (Chen et al., 2004; Falciatore and Bowler, 2005; Christie, 2007; Heijde and Ulm, 2012). Genome sequencing of the seagrass species, *Zostera marina*, reported a loss in three of the five phytochromes. However, sequences for both PHYA and PHYB phytochromes (often associated with seed germination and several other red light responses in terrestrial angiosperms) were present (Olsen et al., 2016). An alternative explanation is that the different responses between different seed experiments were a response to the use of different PPFD and saturating light, so further investigation into specific wavelengths of light is required.

The aim of this study was to determine whether *P. australis* responds to specific wavelengths of light (light quality) and whether any responses are consistent across adult and seedling life history stages. The null hypotheses were: (1) adults and seedlings exposed to different monochromatic wavelengths of light would show no differences across a range of physiological, morphological and biomass measures compared to those grown in a full-spectrum treatment; and (2) there were no differences in the responses of adult and seedling plants.

## Materials and Methods

### Experimental Design and Set-up

In two experiments, the influence of monochromatic light treatments were tested on *P. australis* adults and seedlings separately with the single fixed factor ‘Light quality’ provided at five levels: blue (peak λ = 451 nm); green (peak λ = 522 nm); yellow (peak λ = 596 nm) and red (peak λ = 673 nm) wavelengths and a control of full-spectrum light (λ = 400 – 700 nm). Each treatment was standardized to the same amount of photons (200 μmol photons m^-2^ s^-1^). Transmission spectra for all of the treatments and full spectrum are displayed in Supplementary Figure S1 and Supplementary Table S1. For each level, four replicate aquarium tanks (54 L) were established (total *n* = 20 independent glass tanks). Light treatments were randomly allocated to tanks, and each tank was isolated from the others using PVC boards and shade cloth to ensure no leakage of light from surrounding treatments. Sediments were added to the bottom of each tank to a depth of 10 cm with unsorted washed quartz river sand containing (1.3%) shredded seagrass wrack to stimulate microbial activity and natural nutrient availability (Statton et al., 2013; Fraser et al., 2016). Tanks were then filled with seawater (salinity 35). The water in each tank was re-circulated through an individual sump with a pump and filter (300 μm foam block) ensuring each replicate tank was independent. Water temperature and salinity were monitored every 2 days using a conductivity meter (WTW^TM^) and the temperature maintained at 20 – 21°C, and salinity within 35–36.

**FIGURE 2:**
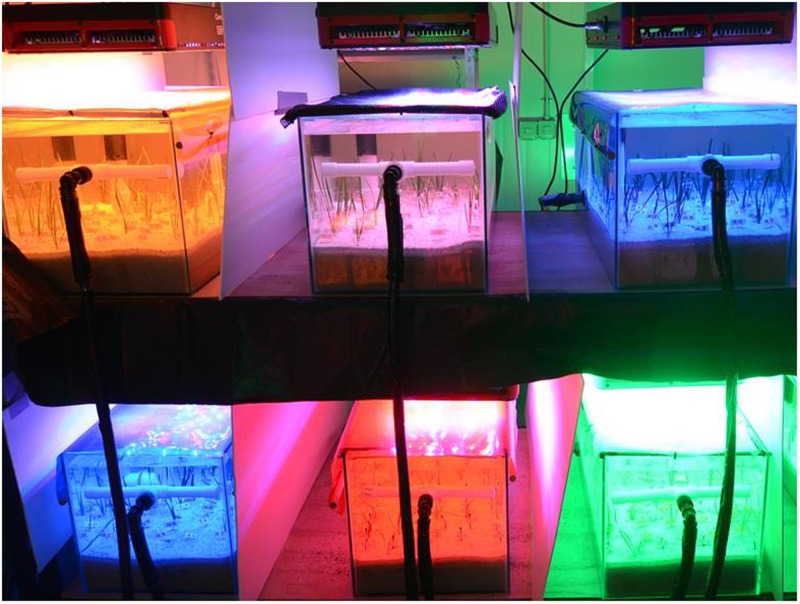
*Posidonia australis* adults and seedlings were grown under yellow **(top left)**, full-spectrum **(top middle)**, blue **(top right)**, red **(bottom middle)** and green **(bottom right)** light in independent aquarium tanks, with four replicates per treatment (*n* = 20 tanks, but only 6 are shown). This image illustrates the seedling experimental set up, though the same was used for the adult experiment (image by Paul Armstrong).

Light treatments were provided through aquarium Light Emitting Diode (LED) Grow8^TM^ modules (MarinTech Pty Ltd., ACT, Australia) customized to a spectrum similar to sunlight set on a 12 h light/dark cycle at 200 μmol photons m^-2^ s^-1^ consistent across all treatments (as measured at the sediment surface using a MicroPAR quantum sensor from In-situ Marine Optics Pty Ltd., Bibra Lake, WA, Australia). This experiment was carried out using the same aquaria set-up (light filters, aquarium lights, sediment type, display tanks, sump tanks and foam block filters) as described in (Strydom et al., 2017). Full-spectrum tanks received light directly from the LED modules and light quality treatments were imposed by placing yellow, red and blue color filters underneath light modules (Rosco heat resistant gel filter sheets, **Figure 2**). For the green treatment, aquarium lights containing all green LED’s were used, as the PPFD quantity could not be achieved using a filter.

### Seagrass Collection and Acclimation

#### Seedlings

Mature *P. australis* fruits (see Statton et al., 2013 for maturity assessment methods) were collected by hand in December 2014 at Garden Island, Western Australia (WA) (32°15′78″E, 115°70′00″S), a temperate shallow meadow (3 m depth) where *Posidonia* spp. dominate. Multiple *P. australis* fruits develop at the top of the peduncle (15 – 60 cm long), with one seed developing per fruit. Fruits are buoyant, and the seeds lack dormancy and are direct developers and are already germinating when fruits dehisce, and the seeds sink to the seafloor (Kuo, 2011). At the time of collection the salinity was 36 and water temperature was 18°C. Fruits were placed into a large holding tank with aerated seawater (temperature: 20°C; salinity: 35) for 1 week allowing them to dehisce seeds under ambient light (Statton et al., 2013). Individual seeds were weighed and then transferred to individual experimental treatment tanks, with 50 individuals placed ∼1 cm below the sediment surface per tank and light treatments applied.

#### Adults

In January 2016, adult *P. australis* ramets were collected from Woodman Point, WA, Australia (-32.13331, 115.74487), from a mixed species meadow at 2 m depth containing *P. australis* with *P. sinuosa, H. ovalis* and *Syringodium isoetifolium.* At the time of collection, the salinity was 35 and water temperature was 19°C. Ramets were collected at haphazard locations along the edge of the meadow by gently excavating the sediment and placed into a cooler box filled with seawater for transportation. Each ramet had at least four shoots preceding an apical meristem. On returning to the laboratory, five ramets were randomly assigned to each aquarium tank: the number of shoots and leaves per shoots counted, all roots removed (as per standard *P. australis* experimental methods, see Hovey et al., 2011) and then planted so that the rhizomes were entirely covered with sediment. In each tank, all leaves in all shoots of a single ramet (referred to as the acclimation ramet) were hole-punched as per standard methods (Short and Duarte, 2001) to determine the leaf extension rate during the acclimation period. Acclimation under full-spectrum light conditions (full-spectrum λ 400 – 700 nm) occurred for 2 weeks. Photosynthetic measurements were used as an indicator of stress to compare aquarium and field plants. The maximum quantum yield values measured at the end of the acclimation period in both aquarium and field plants were in the range identified as healthy, 0.73 – 0.75 (Ralph and Burchett, 1995). This suggested that the plants had successfully acclimated to the aquarium conditions and the acclimation ramet was removed to determine leaf extension rate during the acclimation period. At this point, all leaves of the four remaining ramets per tank were hole punched in order to determine leaf extension rates over the experimental period and light treatments applied for 8.5 weeks, long enough to detect a response in this slow growing species (Hovey et al., 2011).

#### Experimental Measurements

##### Seedling sampling

After 2 weeks of receiving light treatments, five seedlings per tank were removed and processed for analysis. Seedling removal and preparation was repeated for the subsequent 2 weeks and then fortnightly for a total of 14 weeks in order to assess changes in biomass over time and across treatments. At the end of the seedling experiment (14 weeks), the remaining seedlings were harvested and stored at -20°C prior to processing for productivity, morphology, and biomass measures, with the exception of samples for pigment and carbohydrates analysis, which were stored in the dark at -80°C prior to processing.

##### Seedling productivity, biomass and morphology

In the laboratory, all seedlings from each time period were rinsed in seawater, photographed and the number of leaves and roots counted per seedling. Each seedling was then separated into seed, roots, leaves and rhizomes, dried at 60°C for 48 h and weighed to determine total biomass per tank and leaf and root production (g DW d^-1^). Of these, three seedlings from the end of the experiment were used to assess biomass metrics compared among treatments and images of two seedlings per tank were later used to measure leaf area (cm^2^), leaf length (cm), shoot production (shoot d^-1^), and root length (cm) in the program Image J©.

##### Adult productivity, biomass and morphology

Individual adult ramets were photographed on a white background, and the images were used to determine leaf area (cm^2^), number of new leaves and root length (cm) in the program Image J© (**Table 1**). Following photography, the ramets were rinsed in seawater, scraped free of epiphytes and sorted into leaves, living and old leaf sheaths (=above-ground material) and rhizomes plus roots (=below-ground material), and the new leaf extension measured (cm) for each shoot from the base of the leaf sheath to the hole punch. This material was dried at 60°C for 48 h and each component weighed. Productivity (leaf, rhizome and root; mg DW day^-1^) was calculated by summing the weight of all newly produced plant material per tank, divided by the number of days of the experiment and total biomass was the sum of all plant material per tank (mg DW).

**Table 1:** Dependent variables measured at the end of the adult and seedling experiments.

Variable	Measure per tank for seedlings	Measure per tank for adults
**Photosynthetic characteristics**	–	2 mature leaves from different ramets (T_0_ and T_E_)
α, E_k_, ETR_max_, AF, Fv/Fm		
**Pigments**	2 seedlings (T_E_)	2 mature leaves from different ramets pooled (T_E_)
Chlorophyll, xanthophylls		
**Carbohydrates**	2 seedlings pooled into 1 replicate (T_0_, T_E_)	Leaves and rhizomes pooled from 3 ramets into 1 replicate (T_E_)
Adult leaves and rhizomes Seedling leaves and seeds		
**Biomass**	3 seedlings (T_0-_T_E_)	All 4 ramets each with 4 shoots (T_E_)
Total, leaf, rhizome and roots Total seedling, leaf, rhizome, root		
**Leaf characteristics**	2 seedlings (T_E_)	All 4 ramets each with 4 shoots (T_E_)
Leaf number, area, length		
**Root characteristics**	2 seedlings (T_E_)	All 4 ramets each with 4 shoots (T_E_)
Root number, root length, lateral root length and number		


*T_*0*_, end of acclimation and T_*E*_, end of the experiment.*

##### Carbohydrate analysis

Carbohydrates were assessed to determine if energy storage varied across treatments. For the seedlings, carbohydrate analysis was performed on one leaf and one endosperm sample (remaining seed) per tank (each sample being formed by pooling two individual seedlings from each tank) at the end of the experiment. For the adults, carbohydrate analysis was performed on leaf and rhizome material pooled from three ramets per tank. In both cases, dried material (60°C for 48 h) was homogenized and ground into a fine powder in a mill grinder (Mixermill Germany). Seedling seeds, seedling leaves, adult leaves and rhizomes were analyzed separately for soluble sugars and starch content and total carbohydrates using enzymatic procedures adapted from McCleary and Codd (1991).

##### Photo-physiology

A range of photo-physiological characteristics were measured to indicate how the transport of electrons and photosynthetic efficiency varied among plants growing under different light treatments. At the end of the adult experiment (58 days) and prior to the final harvest, photosynthetic characteristics were measured using a Diving Pulse Amplitude Modulated (PAM) fluorometer (Walz Germany). Rapid light curves (RLC) were performed on two mature leaves per tank immediately after the leaf clip was secured, and exposed leaves to increasing PPFD values (1, 11, 34, 64, 115, 176, 287, 415, 670 μmol photons m^-2^ s^-1^ for 10 s) (Ralph and Gademann, 2005). The absorption factor (AF) for each leaf was determined following Beer and Björk (2000) and Electron Transport Rates (ETR) were calculated following the standard protocol (Beer et al., 2001). ETR-Irradiance were fitted to the equation described by Jassby and Platt (1976) to estimate ETR_max_, photosynthetic efficiency (α) and saturating irradiance (E_k_) using SigmaPlot (version 7). Additionally, the maximum quantum yield was measured on two separate mature leaves per tank.

##### Pigment analysis

To determine if changes in light quality induced a level of adjustment at the physiological level, as alterations in pigment content indicate acclimatory responses, both primary and accessory pigments were measured. Chlorophyll *a* and *b* (μg pigment g^-1^ FW leaf tissue) and accessory pigments lutein, β,β carotene, neoxanthin, violaxanthin, zeaxanthin, and antheraxanthin content were analyzed on seedling leaves from two separate seedlings (analyzed as individual replicates) per tank. For adults the same pigments were analyzed for a mid-section of newly produced leaf from two different ramets pooled into one replicate per tank. Leaves were wrapped in foil, placed on dry ice and stored at -80°C for 1 month prior to pigment extraction using the method described in Collier et al. (2008). Supernatants were analyzed using high performance liquid chromatography (HPLC) comprised of a 600 controller, 717 plus refrigerated autosampler and a 996 photodiode array detector (Plazaola and Esteban, 2012). A β,β carotene standard was run through the HPLC machine and values were within the acceptable range. Chlorophyll concentrations were determined using a spectrophotometer and equations based on Wellburn (1994).

#### Statistical Analyses

A multivariate approach was taken to analyze the effect of light quality (fixed factor) on the response of adult plants and seedlings using PRIMER v7 and PERMANOVA+ 2015 (PRIMER-E, Plymouth, United Kingdom), as per Strydom et al. (2017). Separate tests were used for adult and seedling experiments. To illustrate the differences among treatments a metric multidimensional scaling (*m*MDS) plot (Kruskal, 1964) was created and the average for each treatment with an 95% confidence interval was plotted using the Bootstrap Averages routine (Clarke and Gorley, 2015). For each experiment, the response variables measured at T_E_ as identified in **Table 1** were included and normalized. Each of the 5 treatments was assigned 4 replicate tanks (*N* = 20) and each tank contained 4 ramets that were subsequently monitored and measured. Each tank yielded a single measurement for each variable generated either by pooling multiple ramets and/or shoots or by averaging the data from multiple ramets and/or shoots for the adult experiment or by pooling or averaging the data from multiple seedlings for the seedling experiment. A test for homogeneity of variance was performed (PERMDISP) and a permutational analysis of variance (PERMANOVA) run on the resemblance matrix (created using Euclidean distance). A separate PERMANOVA was conducted on the time step seedling biomass data to assess any differences among treatments over time. Where PERMANOVA indicated a significant main effect, a permutational pair-wise test was performed to determine which levels of treatment were significantly (*p* < 0.05) different to each other. As we were interested in determining which of the response variables were contributing most to the differences between light treatment groups, a Similarity Percentage (SIMPER) analysis was performed on the significant pair-wise results with a conservative cumulative % cut-off at 30% and as the amount of variables per group was not excessive, no restriction on the square distance/SD value was enforced. Lastly, univariate PERMANOVAs were carried out on variables identified as important by the SIMPER analysis, to confirm if they were significantly affected by each light quality treatment. The significant differences among treatments for the majority of these variables are displayed in **Figures 4**, **5** (exceptions lutein and violaxanthin for adults as these showed no significantly different patterns and are not discussed in detail in the Discussion), however, the full set of statistical outputs is presented in Supplementary Table S2.

## Results

### Adults

**FIGURE 3:**
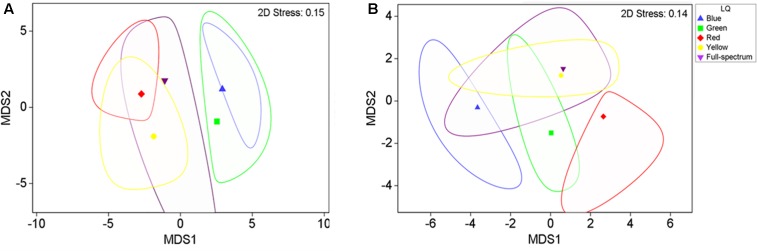
2-D *m*MDS of *P. australis* adult **(A)** and seedling **(B)** samples grown under different light quality treatments (B, blue; G, green; Y, yellow; R, red; FS, full-spectrum). The figure shows the group means surrounded by a corresponding bootstrap region (with 95% confidence interval at 100 bootstraps per group).

The MDS analysis illustrated a clear separation of adult plant samples from the short wavelength treatments (blue and green) from the longer wavelength treatments (yellow and red) along the first axis of the MDS (**Figure 3A**), while the full spectrum samples tended to fall intermediate among the monochromatic treatments.

PERMANOVA analysis confirmed that there was a significant effect of light quality on the characteristics of adult *P. australis* plants (*p* < 0.05) (**Table 2**). However, subsequent pairwise tests indicated that the significant difference lay entirely between the different monochromatic treatments (blue v red, blue v yellow, and green v red treatments differed significantly; *p* < 0.05) and never between the full spectrum treatment and a monochromatic treatment (**Table 2**, pairwise tests). Therefore, the hypothesis that there was no significant difference between full-spectrum and monochromatic light quality treatments was not rejected, and the hypothesis that no significant differences would occur between specific monochromatic light treatments was rejected.

**Table 2:** Results of PERMANOVA test for the effect of light quality (fixed factor) on response variables in the *P. australis* adult and seedling experiments.

Source	d.f.	MS	*F*	Unique perms	*p*
**Main test**					
(1) Adults: Light Quality	4	30.92	1.57	999	<0.05
(2) Seedlings: Light Quality	4	27.07	1.89	999	<0.05
**Pairwise test**					
(1) Adults:
Blue, Yellow				35	<0.05
Blue, Red				35	<0.05
Green, Red				35	<0.05
(2) Seedlings:
Blue, Full-spectrum				35	<0.05
Blue, Red				35	<0.05


*Results are significant if *p* < 0.05. For the post-hoc pairwise tests only the significant interactions are shown.*

**FIGURE 4:**
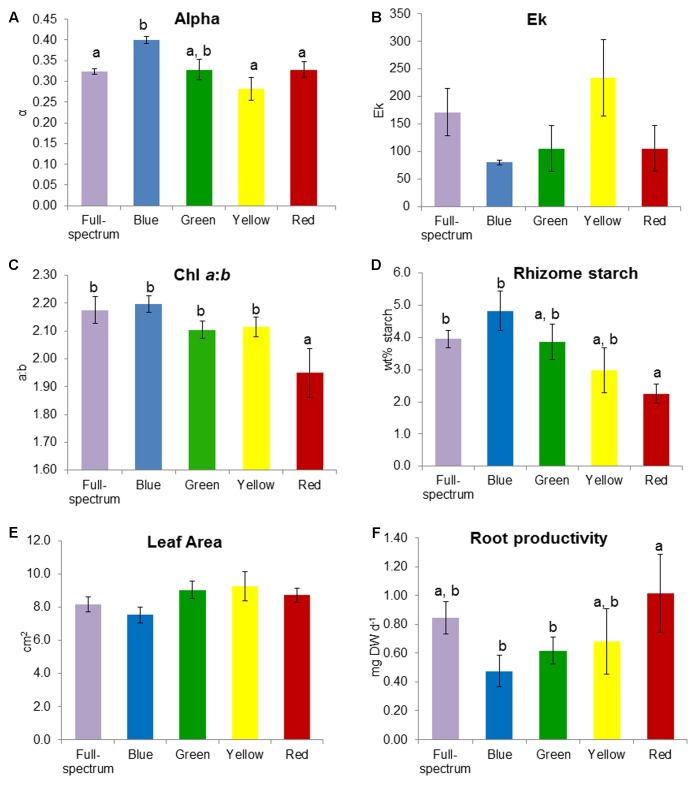
Effect of monochromatic light quality on *Posidonia australis* adult **(A)** photosynthetic efficiency α, **(B)** E_k_, **(C)** chlorophyll *a:b*, **(D)** rhizome starch, **(E)** leaf area and **(F)** root productivity. Different lower case letters denote significant differences (*p* < 0.05) among mean values (±SE) when a significant difference was detected.

SIMPER analysis indicated that the variables contributing to the differences among groups were photophysiological (α, E_k_), pigments (chl *a*:*b*, violaxanthin, lutein) and root productivity (**Table 3**). For the blue v red comparison, plants grown under blue light had higher α, chl *a*:*b* values and rhizome starch compared to those grown under red light, while root productivity was higher in the red light treatment. For the blue v yellow comparison, plants grown under blue light again had higher α values but lower E_k_, rhizome starch and leaf area values compared to in the yellow treatment. For the green v red comparison, plants grown under green light had higher violxanthin, lutein and chl *a*:*b* values compared to those in the red treatment but, as in the blue v red comparison, root productivity was again higher in plants grown in red light. Overall, plants grown under blue light tended to have higher α (compared to those grown in red and yellow) while plants grown under red light tended to have higher root productivity (compared to blue and green). The variables identified by SIMPER as explaining differences between treatments were reassessed using univariate PERMANOVA. Generally, these analyses confirmed that these variables significantly differed to the controls: α was higher in blue, and chl *a*:*b* values and rhizome starch content were lower in red light treatments (**Figure 4**).

**Table 3:** SIMPER summary table indicating *P. australis* response variables that contributed to the observed average distances between the light quality treatments (cumulative % cut-off at 30%).

Variable	Av. Value	Av. Value	Av. Square Distance	Square Distance/SD	Contribution %	Cumulative %
**Adults**	**Blue**	**Red**				
Chl *a:b*	0.699	-1.25	5.39	0.80	10.76	10.76
Rhizome starch	0.83	-0.988	4.36	0.91	8.71	19.47
Root productivity	-0.679	0.78	0.78	0.73	8.00	27.47
Alpha	1.36	-0.343	3.35	1.40	6.69	34.16
	**Blue**	**Yellow**				
Alpha	1.36	-0.899	5.97	1.40	11.24	11.24
Rhizome starch	-0.607	0.809	5.24	0.62	9.87	21.11
E_k_	-0.748	0.926	4.51	0.74	8.49	29.60
Leaf area	-0.845	0.594	4.02	0.91	7.58	37.18
	**Green**	**Red**				
Violaxanthin	0.968	-0.83	4.46	0.97	9.55	9.55
Lutein	0.873	-0.821	3.80	1.14	8.13	17.68
Chl *a:b*	-0.026	-1.25	3.08	0.64	6.59	24.28
Root productivity	-0.285	0.73	2.97	0.68	6.36	30.64
**Seedlings**	**Blue**	**Full-spectrum**				
Carotene	1.69	-0.584	5.70	1.72	12.87	12.87
Lutein	1.51	-0.657	5.68	1.15	12.82	25.69
Neoaxanthin	1.44	-0.732	5.64	1.36	12.72	38.41
	**Blue**	**Red**				
Total biomass	-1.01	1.19	5.69	1.36	9.79	9.79
Carotene	1.69	-0.516	5.22	2.00	8.97	18.76
Leaf productivity	-0.304	1.63	4.4	1.32	7.57	26.33


### Seedlings

**FIGURE 5:**
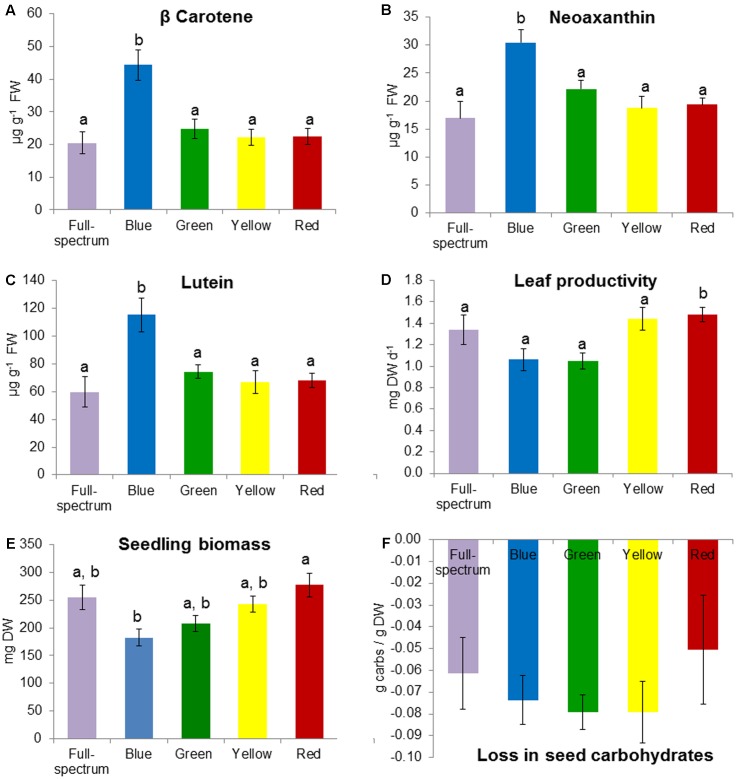
Effect of monochromatic light quality treatments on *Posidonia australis* seedlings: **(A)** carotene, **(B)** neoaxanthin, **(C)** lutein, **(D)** leaf productivity, **(E)** total seedling biomass at end of experiment and **(F)** loss in seed carbohydrates between T_0_ and T_E_. Different lower case letters denote significant differences (*p* < 0.05) among mean values (±SE) when a significant difference was detected.

The blue and red treatments diverged from the full-spectrum treatment in opposite directions along the first axis of the MDS, while the green and yellow treatments clustered with control (**Figure 3B**). For *P. australis* seedlings, the effect of light quality was significant (*p* < 0.05) (**Table 2**). The hypotheses that there were no significant differences between full-spectrum and monochromatic treatments, and no significant differences between monochromatic light quality treatments, were rejected: plants growing under blue light were significantly (*p* < 0.05) different to the full-spectrum and red treatments (**Table 2**). For seedlings, as for adults, the SIMPER analysis indicated that the variables contributing to the difference between blue and red groups included a pigment (in this case, carotene) and productivity but, unlike adult plants, also biomass. Carotene concentrations were higher, while leaf productivity and total biomass were lower in the blue treatments compared to red (**Table 3**). Seedlings grown in blue light also differed to full-spectrum, with higher concentrations of several pigments (**Figure 5**). Univariate PERMANOVAs performed on the seedling variables from the SIMPER analysis showed that seedlings grown in blue light had significantly (*p* < 0.05) higher carotene, lutein and neoxanthin concentrations compared to full-spectrum, and seedling biomass was significantly (*p* < 0.05) lower in blue compared to red treatments (**Figure 5**). Leaf productivity was also significantly higher in red compared blue, but for this variable, it was also significantly higher compared to all other treatments (**Figure 5D**). The amount of total carbohydrates depleted from seeds was not significantly different across treatments at the end of the experiment (**Figure 5F**). Seed biomass declined over time but there were no significant differences between treatments (Supplementary Table S3).

## Discussion

*Posidonia australis* adults did not respond to changes in light quality under high irradiance, where a response is defined as a difference compared to full-spectrum light. Instead our findings demonstrated that this species has a capacity to effectively utilize a number of wavelengths across the visible spectrum to maintain short-term growth. We suggest that the 200 μmol photons m^-2^ s^-1^ that the adult plants were receiving in all monochromatic treatments was saturating, i.e., sufficient to maintain photosynthesis and productivity at levels not significantly different to full-spectrum. Our experiment was performed over 9 weeks and it is possible that over a longer period adult plants might show a response. Nonetheless our findings indicate that this persistent species can resist changes in light quality for short periods of time (i.e. <9 weeks). Seedlings also survived under all of the monochromatic light treatments, but did show some responses: under blue light, seedling pigments were increased and photosynthetic processes modified.

**FIGURE 6:**
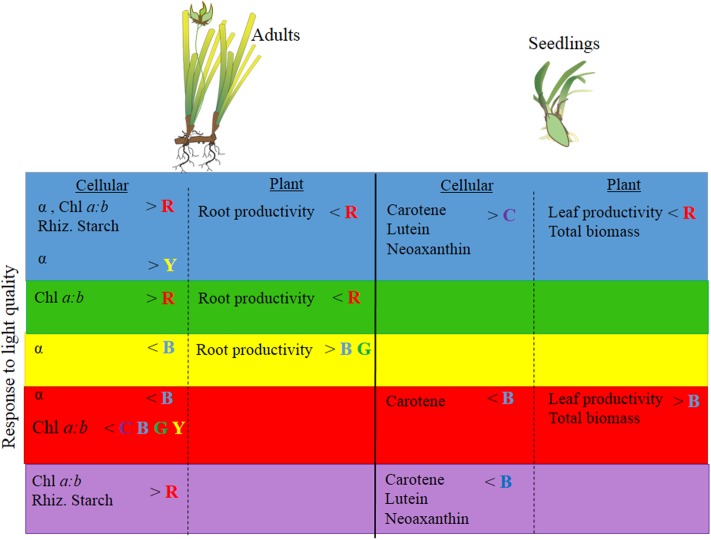
Summary of *P. australis* adult and seedling physiology, productivity and biomass responses (based on univariate PERMANOVA results) to blue, green, yellow, red and full-spectrum (depicted as purple) treatments. *P. australis* images adapted from those created by Catherine Collier, Integration and Application Network, University of Maryland Center for Environmental Science (http://ian.umces.edu/imagelibrary). No permission is required from the copyright holders for its reproduction.

While full-spectrum light provided a useful control condition for this experimental study, it does not exactly reflect the light that seagrasses receive *in situ* (e.g., **Figure 1**). Seagrasses rarely grow under full spectrum light conditions. It is possible that a plant could be growing under predominantly blue light conditions and then be exposed to predominantly red light. In such a scenario, the responses we observed among different monochromatic light treatments become informative. In adults and seedlings we would expect changes in pigment concentrations and increased productivity, while in seedling we would also expect, increased biomass (**Figure 6**). Therefore, environmental conditions that affect light quality (particularly those that simultaneously reduce PPFD) by reducing red light *in situ* could affect *P. australis* depending on the life history stage of the seagrass present.

### *Posidonia australis* Adults and Seedlings Respond to Different Wavelengths of Light

The main outcome from this work is the demonstration that *P. australis* adults were able to adjust their photosynthetic processes and maintain photosynthetic rates and growth, even under narrow wavebands of light. This capacity to acclimate to the extreme ends of the visible spectrum at saturating irradiance implies that the amount of light received is more important than the specific wavelengths of light it receives. The lack of severe response to extreme changes in light quality was not entirely surprising. Since evolving back into the oceans ∼85 million years ago (Les et al., 1997), seagrasses have been subjected to an environment in which a variety of processes alter the wavelengths of light reaching the seafloor. Consequently, the ability to maintain productivity under a wide variety of light quality conditions would confer an adaptive advantage to seagrasses. While our study only demonstrates this potential over a timescale of several weeks, most natural processes which affect light quality (e.g., floods, cyclones, algal blooms) tend to occur at those timescales or shorter (Longstaff and Dennison, 1999; Moore and Jarvis, 2008). The implications of this outcome are that *P. australis* is unlikely to be severely impacted in the short-term by activities and processes that alter light quality (i.e., dredging, algae blooms) without significant reductions in PPFD (below 200 μmol photons m^-2^ s^-1^). However, the majority of these light altering processes tend to simultaneously reduce PPFD and previous work has demonstrated that *Posidonia* spp. have physiological, morphological and growth responses to reduced PPFD. For example, *P. sinuosa* showed an 82% decline in shoot density when grown at 14 μmol photons m^-2^ s^-1^ for 3.5 months (Collier et al., 2009). Therefore, impacts of altered light climates are likely when *Posidonia* receives PPFD below its minimum light requirement (thereby reducing its effectiveness in being able to acclimate) and for periods of time greater than 3 months (i.e., beyond the experimental period of the experiments in this study).

Seedlings exhibited photo-physiological alterations to pigment content by significantly increasing xanthophyll concentrations under blue light (compared to full-spectrum). This demonstrates a physiological plasticity in seedlings, allowing them to survive and grow under a range of light conditions. This has been found in a number of terrestrial angiosperms, where increased carotenoid concentrations were detected in leaves growing under blue light (Lee et al., 2007), which potentially enhances photosynthetic rates as these pigments absorb blue light and transfer the energy to chlorophylls. The seedling pigment response could indicate an ability for seedlings to establish in deeper waters, where blue light dominates. Why adults did not increase xanthophyll concentrations in response to blue light as well is not clear; it is possible that any physiological plasticity is a developmental trait that is absent in the adult plants or adults can rely on carbohydrate storage reserves to resist changes in light quality.

### Physiological and Productivity Variables Differed between Blue and Red Treatments

Adults demonstrated physiological changes when grown under some of the different light wavelength treatments, with no significant effect on biomass. The increased photosynthetic efficiency and chl *a*:*b* under blue light compared to red light are likely due to differences in energy output of short and long wave radiation. Blue light has a higher frequency and, therefore, more energy per photon compared to red light that can lead to increased photosynthetic efficiency. Despite the increased photosynthetic efficiency in blue light, there were no increases in ETR_max,_ possibly due to the additional energy being dissipated through NPQ (Sun et al., 1998), and this is consistent with the observed lack of effect on biomass. In terrestrial angiosperms blue light has similarly been shown to induce changes in pigment content and ratios, but with no significant effect on biomass (Lee et al., 2007; Mizuno et al., 2011; Alvarenga et al., 2015; Hoffmann et al., 2015). In particular, increased chl *a*:*b* in leaves of terrestrial angiosperms grown under blue light compared to red (Lee et al., 2007) is also indicative of excess energy arising from phytochrome excitation being discharged in chlorophyll *b* (Van Huylenbroeck et al., 2000). Unlike their terrestrial counterparts, seagrasses grow in an environment where they are exposed to predominantly blue light due to the high attenuation of long wavelength radiation by water. Therefore *P. australis* adults, receiving predominantly blue light (i.e., in deep-water environments) are likely to adjust physiologically and assuming the intensity of light is sufficient, this may not necessarily lead to significant changes in biomass compared to those growing in red light-dominated environments in shallow areas.

The increased below-ground productivity of adults and leaf productivity of seedlings with exposure to red light relative to blue reflects similar responses in terrestrial plants (Moon et al., 2006; Lee et al., 2007; Forster and Bonser, 2009; Baque et al., 2011) and may confer advantages to seagrasses in some marine environments. For seagrasses, experiencing a habitat with a large proportion of red light will only occur in shallow water as these wavelengths are strongly attenuated with depth. *P. australis* seedlings are known to establish in such shallow habitats (Ruiz-Montoya et al., 2015). As seagrasses would be subjected to greater swell and hydrodynamics in shallow waters compared to deep waters, increasing root productivity could be a potential ecological advantage to aid in anchorage of adult plants under these conditions. Conversely, the reduced seedling leaf productivity and biomass under blue light suggests that in deeper water, seedlings are likely to grow slower compared to those in shallow waters. In terrestrial plants, responses to red light are mediated through photoreceptors. Photoreceptors have been identified in one seagrass species, *Z. marina* (Olsen et al., 2016). Further study of photoreceptors may provide a way forward in understanding the responses of seagrasses to altered light climates.

### Comparisons between *H. ovalis* and *P. australis*

Based on the findings of this study and an earlier study on *Halophila ovalis* (Strydom et al., 2017), a colonizing seagrass species, *H. ovalis* is more susceptible to light quality changes compared to *P. australis. Posidonia australis* adults did not respond to changes in light quality between monochromatic and full-spectrum light treatments, whereas in *H. ovalis*, negative impacts to biomass and below-ground productivity were observed under some monochromatic treatments. The contrasting responses of the two species to altered light quality is generally consistent with Kilminster et al.’s (2015) model of persistent vs. colonizing life-history strategies. Under that model, *P. australis* relies on resistance traits such as slow growth and utilization of carbohydrate stores (which are substantially larger compared to colonizing species) to survive periods of environmental stress, whereas *H. ovalis* is less resistant (i.e., high mortality, reduced growth and biomass) but can recover quickly from disturbance from rapid growth of the remaining vegetative fragments or seed banks.

The negative effect of blue compared to red light on *P. australis* was generally consistent with that observed in *H. ovalis* adults, seeds and seedlings. *Posidonia australis* seedlings had reduced biomass and leaf productivity under blue light compared to red, and *H. ovalis* seed germination and subsequent seedling survival were also significantly lower in the blue treatments compared to red (Strydom et al., 2017), suggesting a common underlying mechanism for these responses in both species. Again, it is possible that photoreceptors could play an important role in any such mechanism.

The lack of significant response between full-spectrum and yellow and green light for both *P. australis* adults and seedlings differed to the significant reduction in below-ground productivity of *H. ovalis* to these wavelengths (Strydom et al., 2017). This may reflect differences in the life-history strategies and environments in which the two species occur. *H. ovalis* often grows in environments subject to dramatic changes in water quality that are unfavorable and in those conditions typically dies back and recovers from seed banks (Rasheed, 2004). Environmental conditions that cause green-yellow shifts in benthic light quality include suspended sediment plumes, i.e., from cyclones, river discharge, dredging etc. (Gallegos et al., 2009; Jones et al., 2016). Such plumes simultaneously reduce PPFD and, as such, even though our results indicate that green and yellow wavelengths may not have negative impacts on *P. australis*, reductions in PPFD certainly do (Ralph et al., 2007; McMahon et al., 2013).

## Conclusion

This study has demonstrated that the seagrass *P. australis* has the capacity to survive under spectra of various wavelengths of light across multiple life history stages. Furthermore, the amount of light that this seagrass receives appears more important than the wavelengths it receives as it was able to maintain growth at 200 μmol photons m^-2^ s^-1^ across narrow wavebands of the visible spectrum. This suggests that from a management perspective changes in light quality may be less significant for persistent species than for colonizing species such as *Halophila ovalis* which have displayed stronger responses to altered light quality. However, processes that simultaneously alter light quality and reduce PPFD to sufficiently low intensities may impact the characteristics (i.e., carbohydrate reserves) that assist seagrasses to remain resilient against other stressors, and any loss of resilience ultimately reduces the long-term viability of populations (Unsworth et al., 2015). Therefore, future work must be aware of, and contemplate how, *P. australis* might respond to changes in light quality in conjunction with other stressors.

## Author Contributions

SS designed and built the experimental aquarium set up, ran all experiments, processed samples, conducted data analysis and drafted the manuscript. KM and PL substantially contributed to the intellectual content of the manuscript through multiple edits as well as providing regular supervision of SS throughout all experiments. GK and JS provided substantial contributions to the conception and design of the study, as well as interpretation of data and in writing the manuscript. All authors provided final approval of the manuscript to be published.

## Conflict of Interest Statement

The authors declare that the research was conducted in the absence of any commercial or financial relationships that could be construed as a potential conflict of interest.
