# Supplementary material for: Short-term Responses of Posidonia australis to Changes in Light Quality
Source: Front Plant Sci. 2018 Jan 17;8:2224. doi: 10.3389/fpls.2017.02224 (PMC5776106; doi:10.3389/fpls.2017.02224)
Supplement: Supplementary file 1 [file Table_1.docx]

Supplementary material

Table S1: The amount of energy per photon per light quality treatment (based on peak wavelength emission) that *H. ovalis* adults, seeds and seedlings were exposed to during the experiments.

| Light quality treatment | Peak emission λ (nm) | Energy per photon (Joules) |
| --- | --- | --- |
| Blue | 451 | 4.4045 ⋅10^−19^ |
| Green | 522 | 3.8055 ⋅10^−19^ |
| Yellow | 596 | 3.3330 ⋅10^−19^ |
| Red | 673 | 2.9516 ⋅10^−19^ |

Note that energy was calculated as follows:

$$e=\frac{\text{c ∙ h}}{\lambda}$$

Where *e* is energy in joules, c is light velocity (299 792 458 m/ sec), h is Planck’s constant (6.6260695729 ⋅10^−34^) and 𝜆 is Lambda which represents wavelength (nm).

Table S2: Results from the univariate PERMANOVA analysis testing the effect of light quality (one fixed factor) on the *P. australis* response variables from the SIMPER analysis. Results are significant if *p* < 0.05.

| Source | d.f. | MS | F | Unique perms | P |
| --- | --- | --- | --- | --- | --- |
| 1. Adults |  |  |  |  |  |
| **Blue vs. Red**  Alpha  Chl *a:b*  Rhizome starch  Root productivity | 19  19  19  19 | 2.77  2.33  2.30  1.91 | 5.25  3.63  3.54  6.39 | 999  999  999  999 | **< 0.05**  **< 0.05**  **< 0.05**  **< 0.05** |
| **Blue vs. Yellow** |  |  |  |  |  |
| Alpha  E_k_  Rhizome starch  Leaf area  **Green vs. Red**  Chl *a:b*  Root productivity  Lutein  Violaxanthin   1. Seedlings   **Blue vs. full-spectrum**  Carotene  Lutein  Neoxanthin  **Blue vs. Red**  Total biomass  Carotene  Leaf productivity | 19  19  19  19  19  19  19  19  39  39  39  39  39  39 | 2.77  2.09  1.70  1.49  2.33  1.91  308  2.23  4.94  4.08  4.04  2.75  4.94  3.41 | 5.25  1.09  2.09  0.25  3.63  6.39  1.69  3.33  8.99  6.30  6.19  5.15  8.99  9.58 | 999  999  999  999  999  999  999  998  998  999  999  999  999  999 | **< 0.05**  > 0.05  **< 0.05**  > 0.05  **< 0.05**  **< 0.05**  > 0.05  > 0.05  < 0.05  < 0.05  < 0.05  < 0.05  < 0.05  < 0.05 |

Table S3: Results from PERMANOVA analysis testing the effect of light quality and time (two fixed factors) on the change in *P. australis* seed weight over time. Results are significant if *p* < 0.05. “T” indicates seed removal time in the pairwise tests.

| Source | d.f. | MS | F | Unique perms | P |
| --- | --- | --- | --- | --- | --- |
| MAIN TEST |  |  |  |  |  |
| Light quality  Time  Light quality x Time  PAIR-WISE TESTS  T1, T2  T1, T3  T1, T4  T1, T5  T1, T6  T2, T3  T2, T4  T2, T5  T2, T6  T3, T4  T3, T5  T3, T6  T4, T5  T4, T6  T5, T6 | 4  5  20 | 0.13  16.58  0.22 | 0.39  48.26  0.66 | 998  999  999  996  998  999  999  997  997  998  997  997  997  995  995  997  998  998 | > 0.05  **< 0.05**  > 0.05  **< 0.05**  **< 0.05**  **< 0.05**  **< 0.05**  **< 0.05**  **< 0.05**  **< 0.05**  **< 0.05**  **< 0.05**  **< 0.05**  **< 0.05**  **< 0.05**  **< 0.05**  **< 0.05**  > 0.05 |

**Figure**


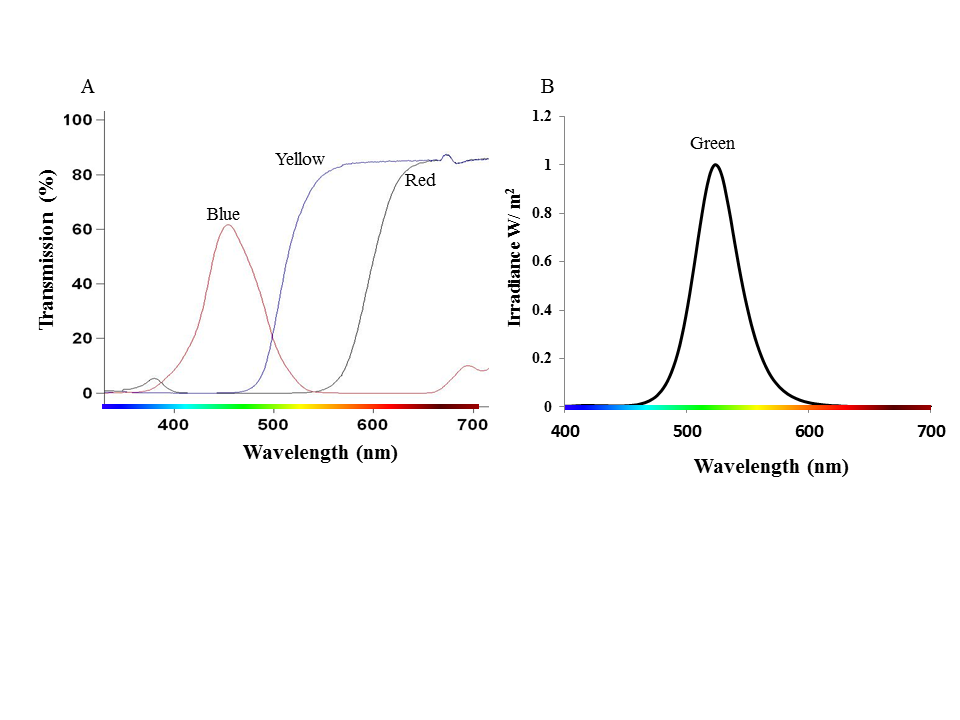
Figure S1. Transmission spectra (%) of the Rosco heat resistant gel filter sheets used to create blue (‘Night Blue’ peak transmission λ=451 nm), red (‘Fire’ peak transmission λ=673 nm) and yellow (‘Canary Yellow’ peak transmission λ=595 nm) light quality treatments (A). The peak spectral output of the LED used to create the green (λ=522 nm) light quality treatment (B).
